# Four collateral circulation pathways were observed after common carotid artery occlusion

**DOI:** 10.1186/s12883-019-1425-0

**Published:** 2019-08-20

**Authors:** Jianan Wang, Chengrong Zheng, Bei Hou, Aihua Huang, Xiongwei Zhang, Bin Du

**Affiliations:** 1 Department of Postgraduate, Third Military Medical University, Chongqing, China; 2 Department of Neurology, General Hospital of the PLA Rocket Force, No.16 Xinjiekouwai Street, Xicheng District, Beijing, 100088 China; 3 Department of Cardiovascular Medicine, General Hospital of the PLA Rocket Force, No.16 Xinjiekouwai Street, Xicheng District, Beijing, 100088 China; 4 Department of Critical Care Medicine, General Hospital of the PLA Rocket Force, No.16 Xinjiekouwai Street, Xicheng District, Beijing, 100088 China; 5 Department of Neurointervention, General Hospital of the PLA Rocket Force, No.16 Xinjiekouwai Street, Xicheng District, Beijing, 100088 China

**Keywords:** Common carotid artery occlusion, Collateral circulation, Internal carotid artery steal

## Abstract

**Background:**

Common carotid artery (CCA) occlusion (CCAO) is a rare condition. Owing to collateral circulation, ipsilateral internal carotid artery (ICA) and external carotid artery (ECA) are often patent.

**Methods:**

This study included 16 patients with unilateral CCAO and patent ipsilateral ICA and ECA. The pathways which supplied ICA were investigated by digital subtraction angiography (DSA), transcranial Doppler (TCD), magnetic resonance angiography (MRA) and computed tomography angiography (CTA).

**Results:**

In all 16 patients, TCD found antegrade blood flow in ipsilateral ICA, which was supplied by retrograde blood flow in ipsilateral ECA through carotid bifurcation. We call this phenomenon “ICA steal”. DSA and CTA discovered four pathways of ICA steal, including 1) ipsilateral vertebral artery – occipital artery – ECA – ICA, 2) ipsilateral thyrocervical trunk or costocervical trunk – ascending cervical artery or deep cervical artery – occipital artery – ECA – ICA, 3) contralateral ECA – contralateral superior thyroid artery – ipsilateral superior thyroid artery – ipsilateral ECA – ICA, and 4) ipsilateral thyrocervical trunk – inferior thyroid artery – superior thyroid artery – ECA – ICA.

**Conclusions:**

ICA is possible to be patent and supplied by several collateral circulation pathways after CCAO.

## Background

One of the risks of ischemic stroke is pathological changes of extracranial carotid circulation. Atherosclerotic occlusion of internal carotid artery (ICA) has been intensively studied for its clinical and imaging characteristics. However, common carotid artery (CCA) occlusion (CCAO) is rarely studied due to its low incidence [1, 2]. CCAO occurs in about 3% of symptomatic patients undergoing angiography [3]. Consensus has not been reached as for the indications, necessities and clinical benefits of invasive treatment of CCAO.

According to Riles et al’s classification, CCAO has four types: type 1A with patent ICA and external carotid artery (ECA), type 1B with occluded ICA and patent ECA, type 1C with patent ICA and occluded ECA, and type 2 with occluded ICA and ECA [4]. According to C. Klonaris et al’s review, most CCAO (61.5%) is type 1A, while type 1B and type 2 account for 26.6 and 11.9%, respectively. Type 1C account for none and may be just theoretical [5].

Since ipsilateral ICA and ECA are often patent after CCAO, there must be collateral circulation to supply them. This collateral circulation may relieve the ischemia of brain and symptoms of patients, thus affecting whether and how CCAO should be treated. So, it is important to discover the pathways of this collateral circulation and to evaluate their compensating abilities. This information, however, is available in few studies [6–8].

Our study was aimed to discover the collateral circulation pathways which could supply ICA after CCAO, and to investigate their hemodynamic and imaging characteristics.

## Methods

We retrospectively examined the medical database of patients who had been admitted to the Department of Neurology or Neurointervention of the PLA Rocket Force General Hospital from January 2013 to December 2015. From the database we found 16 patients with unilateral CCAO but patent ipsilateral ICA and ECA (Riles type 1A). Among them, 9 were male and 7 were female. They were aged between 65 and 83 years old.

We summarized their examination results and images. Transcranial Doppler (TCD) detected the blood flow in CCA, ICA and ECA on both sides. Digital subtraction angiography (DSA), computed tomography angiography (CTA) and Magnetic resonance angiography (MRA) offered dynamic and static images of intracranial and extracranial arteries and collateral circulation. All 16 patients had TCD examination. DSA, MRA and CTA were performed in 15, 5 and 7 patients, respectively (Table 1).

**Table 1:** The 16 patients’ demographic data, risk factors and examinations

Mean age	72.8 (65–83)
Sex	
Male	9
Female	7
Atherosclerotic risk factors	
Smoking	9
Hypertension	14
Hyperlipidemia	9
Diabetes	6
Examinations	
TCD	16
DSA	15
MRA	5
CTA	7

## Results

### TCD

**Fig. 1:**
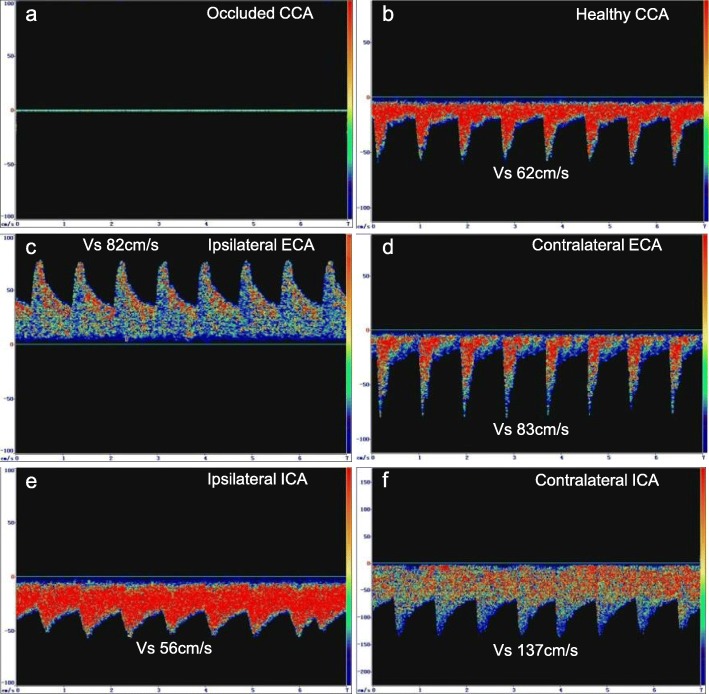
TCD showed the blood flow in CCA, ECA and ICA on both sides of the 16 patients. **a** No blood flow in the occluded CCA. **b** Normal blood flow in the healthy CCA. **c** Retrograde blood flow in the ipsilateral ECA. **d** Normal blood flow in the contralateral ECA. **e** Lower but antegrade blood flow in the ipsilateral ICA. **f** Normal blood flow in the contralateral ICA

No blood flow was detected in the occluded CCA (Fig. 1a), while the healthy CCA had normal blood flow (Fig. 1b). For all 16 patients, retrograde blood flow was detected in the ipsilateral ECA, the spectrum of which was similar to intracranial arteries (Fig. 1c). The contralateral ECA had normal blood flow (Fig. 1d). The ipsilateral ICA had lower but antegrade blood flow (Fig. 1e), while the contralateral ICA had normal blood flow (Fig. 1f). Eight patients had compensatory increased flow velocity in the V3 segment of ipsilateral vertebral artery (systolic velocity 120~150 cm/s, average velocity 80~100 cm/s). These patients also had reversed and increased flow velocity in the affected occipital artery.

### DSA

**Fig. 2:**
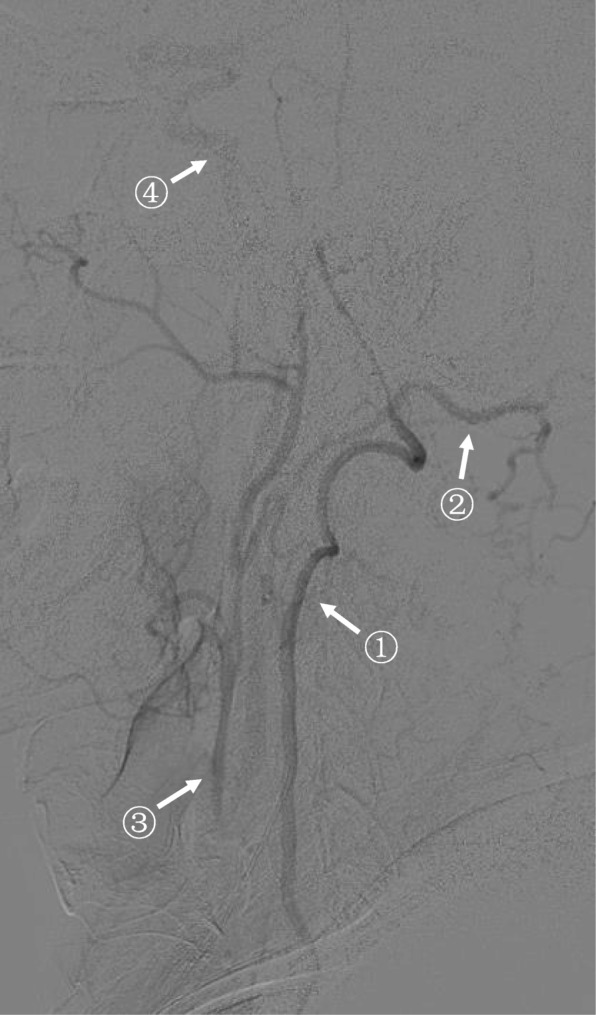
DSA discovered a collateral circulation pathway that supplied ipsilateral ICA after CCAO. This was a right (ipsilateral) subclavian artery angiography (lateral position, arterial phase). Right CCA was occluded. The collateral circulation pathway was: right vertebral artery (arrow^①^) – right occipital artery (arrow^②^) – right ECA (retrograde, arrow^③^) – right ICA (arrow^④^)

**Fig. 3:**
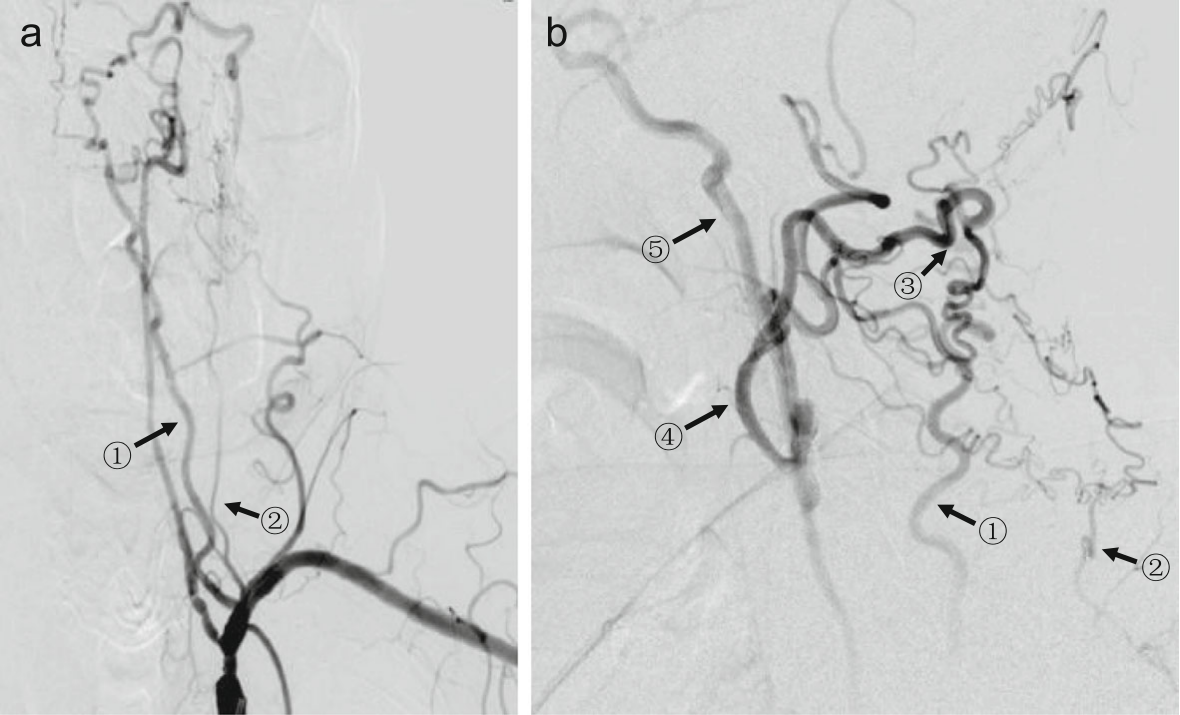
DSA discovered another collateral circulation pathway that supplied ipsilateral ICA after CCAO. This was a left (ipsilateral) subclavian artery angiography (front position (**a**) and lateral position (**b**), arterial phase). Left CCA was occluded. The original part of left subclavian artery had moderate stenosis. The V1 segment of left vertebral artery had severe stenosis. The V4 segment of left vertebral artery became undeveloped after it gave out the posterior inferior cerebellar artery. The collateral circulation pathway was: ascending cervical artery from left thyrocervical trunk (arrow^①^) and deep cervical artery from left costocervical trunk (arrow^②^) – occipital artery (retrograde, arrow^③^) – ECA (retrograde, arrow^④^) – carotid bifurcation – ICA (arrow^⑤^)

**Fig. 4:**
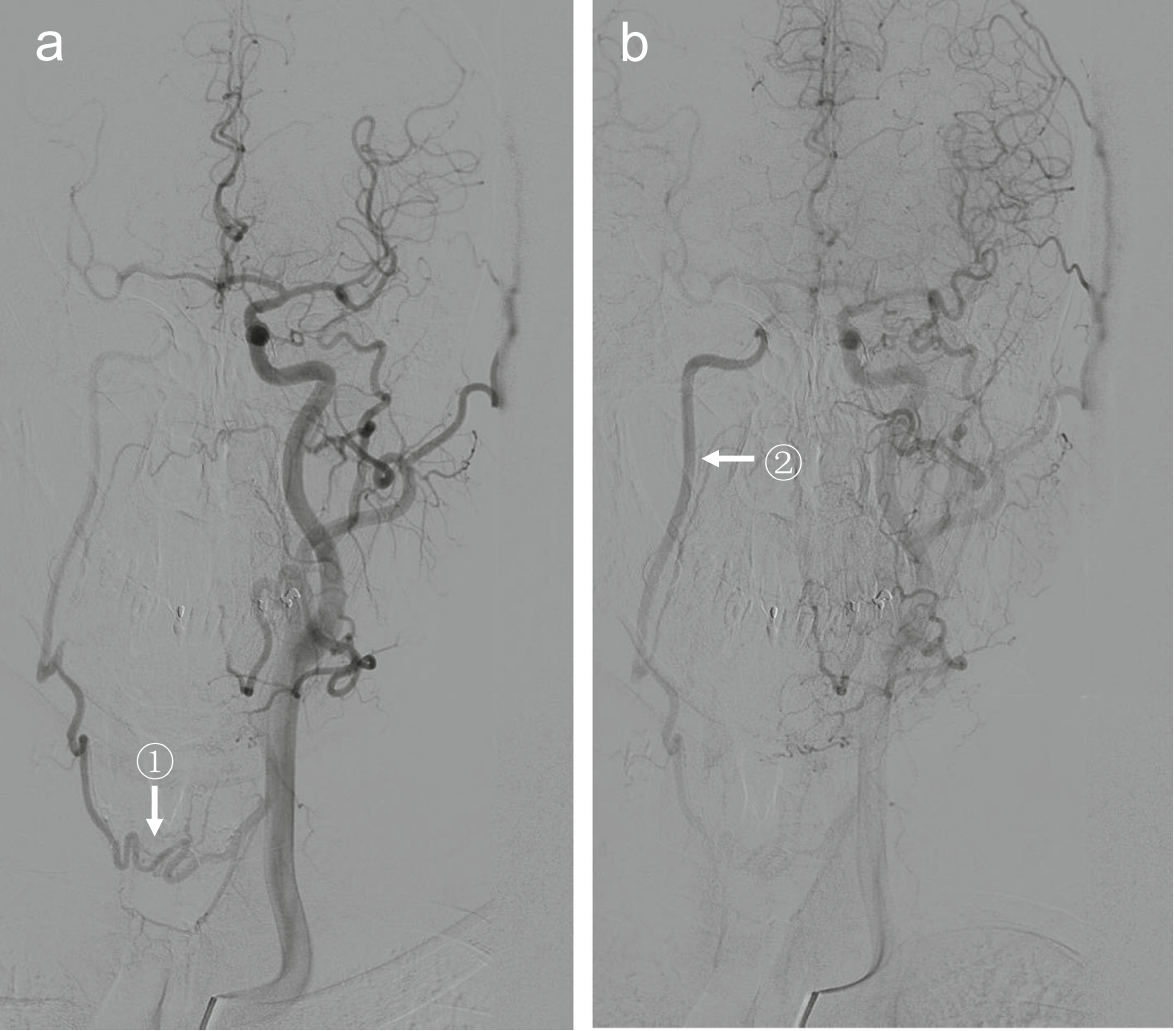
DSA discovered a third collateral circulation pathway that supplied ipsilateral ICA after CCAO. This was a left (contralateral) CCA angiography (front position, arterial phase (**a**) and parenchymal phase (**b**)). Right CCA was occluded. The collateral circulation pathway was: left ECA – left superior thyroid artery – thyroid anastomosis (arrow^①^) – right superior thyroid artery – right ECA – right ICA (arrow^②^)

DSA was performed in 15 of the 16 cases. They all had retrograde blood flow in ipsilateral ECA and antegrade blood flow in ipsilateral ICA. DSA discovered three collateral circulation pathways that supplied ipsilateral ICA. Pathway 1: ipsilateral vertebral artery – occipital artery – ECA – ICA (Fig. 2). Pathway 2: ipsilateral thyrocervical trunk or costocervical trunk – ascending cervical artery or deep cervical artery – occipital artery – ECA – ICA (Fig. 3). Pathway 3: contralateral ECA – contralateral superior thyroid artery – ipsilateral superior thyroid artery – ipsilateral ECA – ICA (Fig. 4). These three pathways existed in 8, 4 and 1 patients, respectively. We failed to find any specific pathways in the remaining 2 patients.

### MRA and CTA

**Fig. 5:**
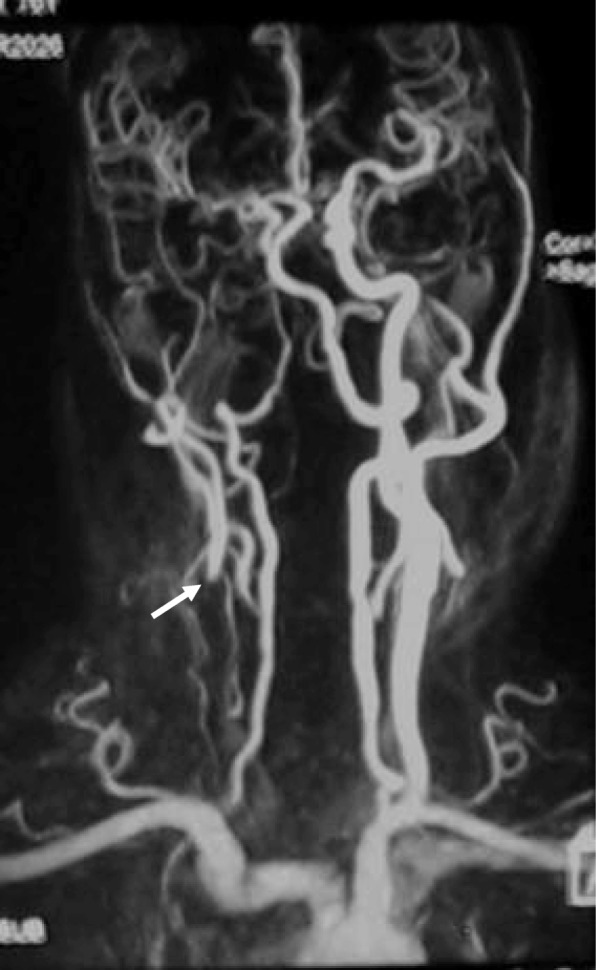
MRA results. MRA showed the occlusion of right CCA (arrow). Right carotid bifurcation, ICA and ECA and were partially developed

**Fig. 6:**
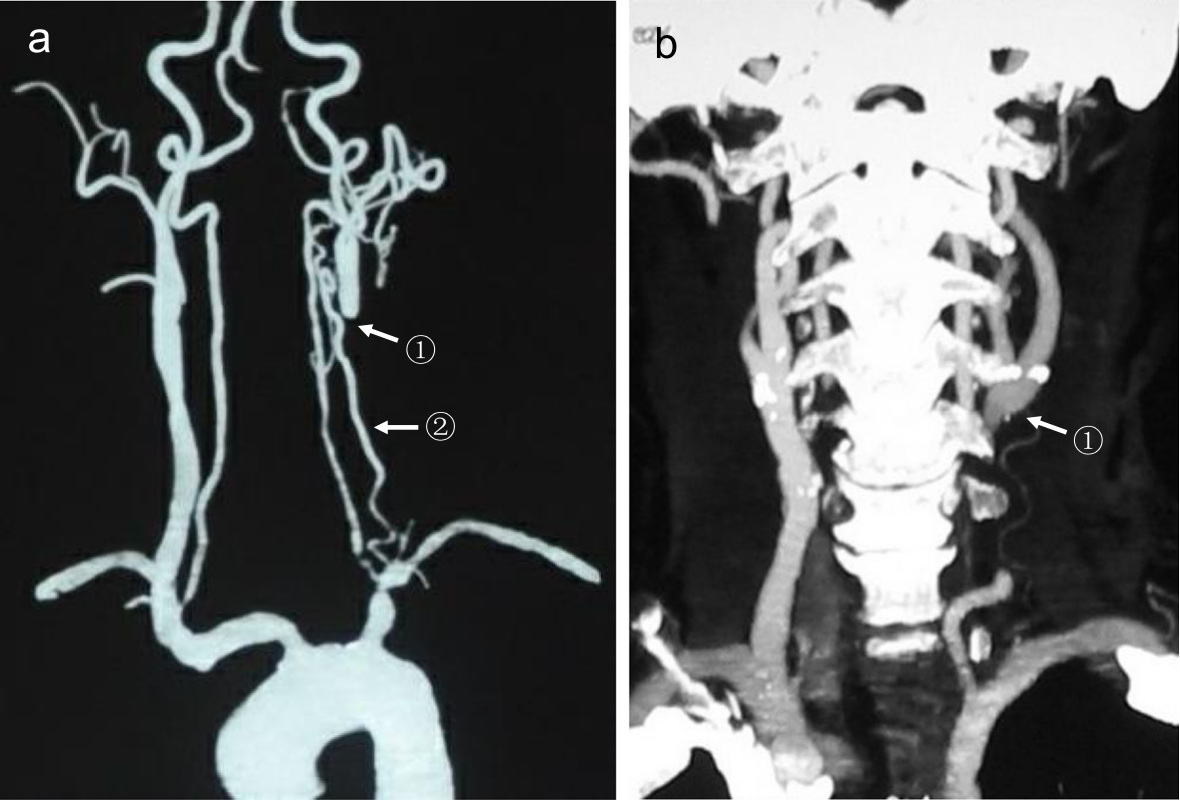
CTA results. CTA showed the occlusion of left CCA. Left ICA, ECA and carotid bifurcation (arrow^①^(**a**,**b**)) were well developed. No abnormality was found in the development of distal arteries. Vertebral artery and thyrocervical trunk (arrow^②^ (**a**)) which participated in collateral circulation were also developed

MRA (Fig. 5) and CTA (Fig. 6) showed occluded CCA but patent ipsilateral ICA, ECA and carotid bifurcation. Vertebral artery and thyrocervical trunk which participated in collateral circulation could also be shown.

**Fig. 7:**
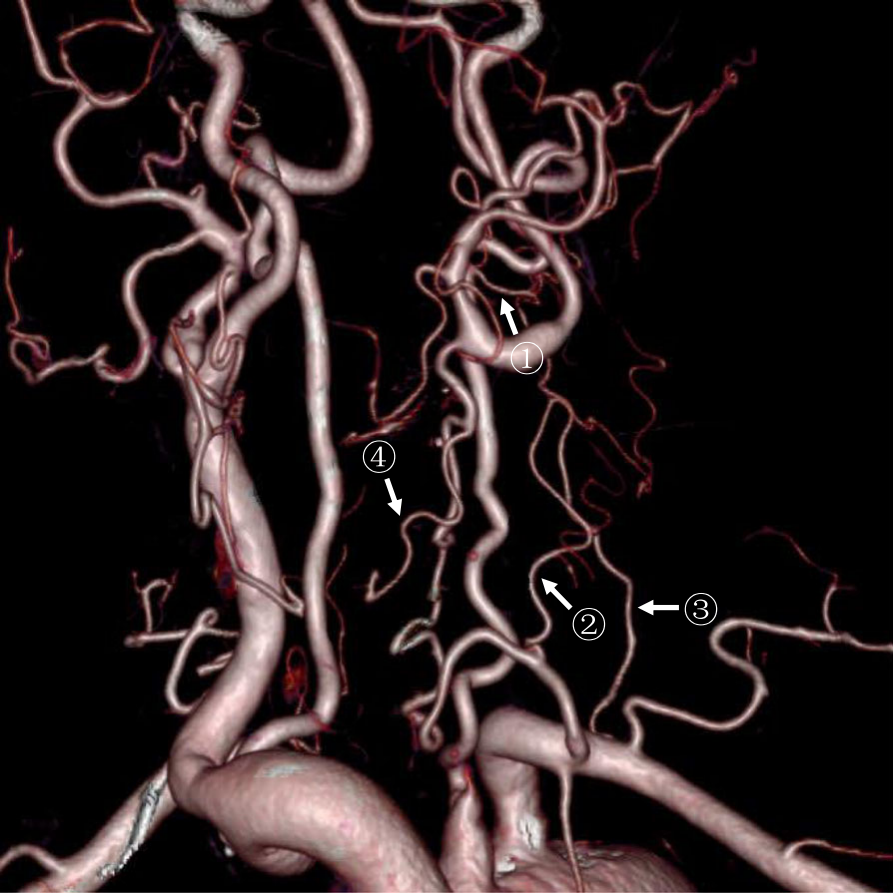
CTA discovered a fourth collateral circulation pathway that supplied ipsilateral ICA after CCAO. CTA showed coexistence of several collateral circulation pathways in one patient. Pathway 1: ipsilateral vertebral artery – occipital artery (arrow ^①^) – ECA – ICA. Pathway 2a: ipsilateral thyrocervical trunk – ascending cervical artery (arrow^②^) – occipital artery – ECA – ICA. Pathway 2b: ipsilateral costocervical trunk – deep cervical artery (arrow ^③^) – occipital artery – ECA – ICA. Pathway 4: ipsilateral thyrocervical trunk – inferior thyroid artery – superior thyroid artery (arrow ^④^) – ECA – ICA

In particular, in the one patient who did not had DSA examination, CTA showed coexistence of the pathways 1 and 2 mentioned above, and a fourth pathway: ipsilateral thyrocervical trunk – inferior thyroid artery – superior thyroid artery – ECA – ICA (Fig. 7).

## Discussion

CCAO is a rare condition that may be associated with ischemic cerebral diseases. There is no clear consensus on the role of invasive treatment of CCAO. Owing to collateral circulation, about 61.5% of patients with CCAO have patent ipsilateral ICA and ECA [5]. For patients with ischemic cerebrovascular diseases, collateral circulation evaluation has great clinical significance, such as in explaining clinical manifestations, developing therapeutic schedule, evaluating the efficacy of treatments and estimating prognosis [9–11].

The basic driving force that leads to the formation of collateral circulation is the change in blood pressure. When a main artery from aortic arch is occluded, the blood pressure in the distal segment can be much lower than adjacent arteries. As a result, blood flow in adjacent arteries may be drawn reversely to the distal segment. This phenomenon is called “steal”, with subclavian steal syndrome as an example. When CCA is occluded, the blood pressure in ipsilateral ICA can be obviously lower than ipsilateral ECA. As a result, ICA “steals” blood from ECA. This phenomenon was defined as “carotid artery steal” by Anne G. Osborn [12]. However, we think this definition is not clear enough. Since ECA supplies blood and ICA receives blood, we propose a new definition here – “ICA steal”.

Our study discovered four collateral circulation pathways of “ICA steal” by DSA and CTA: 1) ipsilateral vertebral artery – occipital artery – ECA – ICA, 2) ipsilateral thyrocervical trunk or costocervical trunk – ascending cervical artery or deep cervical artery – occipital artery – ECA – ICA, 3) contralateral ECA – contralateral superior thyroid artery – ipsilateral superior thyroid artery – ipsilateral ECA – ICA, and 4) ipsilateral thyrocervical trunk – inferior thyroid artery – superior thyroid artery – ECA – ICA. Pathway 4 was previously reported by Lie TA et al. [6]. This collateral circulation may also involve other branches of ECA theoretically, such as facial artery, maxillary artery and superficial temporal artery; but this has not been reported yet.

The imaging characteristics of ICA steal under CTA and MRA include: 1) disappearance of the original segment of CCA, 2) normal development of ICA, ECA and carotid bifurcation, and 3) development of thyrocervical trunk in some patients. The hemodynamic characteristics of ICA steal under TCD include: 1) no blood signal in the occluded CCA; 2) retrograde blood flow in ipsilateral ECA with similar spectrum to intracranial arteries; 3) lower but antegrade blood flow in ipsilateral ICA. These phenomena indicate the possibility of ICA steal.

Our study has some limitations. We did not quantitatively evaluate the influence of these collateral circulation pathways on brain perfusion. In fact, this is crucial since it may affect whether a patient with CCAO needs further invasive treatment. Further studies are needed to evaluate the brain perfusion of different Riles types and different collateral circulation pathways, and to analyse how compensation affects patients’ neurological symptoms, benefits from invasive treatment, and prognosis.

## Conclusion

ICA is possible to be patent and supplied by several collateral circulation pathways after CCAO.

## Data Availability

The data and images used in the current study are available from the corresponding author upon reasonable request.

## Abbreviations

CCA: Common carotid artery
CCAO: Common carotid artery occlusion
CTA: Computed tomography angiography.
DSA: Digital subtraction angiography
ECA: External carotid artery
ICA: Internal carotid artery
MRA: Magnetic resonance angiography
TCD: Transcranial Doppler
